# Regulatory actions of ToxR and CalR on their own genes and type III secretion system 1 in *Vibrio parahaemolyticus*

**DOI:** 10.18632/oncotarget.19498

**Published:** 2017-07-22

**Authors:** George Osei-Adjei, He Gao, Ying Zhang, Lingyu Zhang, Wenhui Yang, Huiying Yang, Zhe Yin, Xinxiang Huang, Yiquan Zhang, Dongsheng Zhou

**Affiliations:** ^1^ School of Medicine, Jiangsu University, Zhenjiang 212013, China; ^2^ State Key Laboratory of Pathogen and Biosecurity, Beijing Institute of Microbiology and Epidemiology, Beijing 100071, China; ^3^ State Key Laboratory of Infectious Disease Prevention and Control, National Institute for Communicable Disease Control and Prevention, Chinese Center for Disease Control and Prevention, Beijing 102206, China

**Keywords:** Vibrio parahaemolyticus, calR, ToxR, T3SS1

## Abstract

*Vibrio parahaemolyticus* is the leading cause of seafood-associated gastroenteritis. Type III secretion system 1 (T3SS1) is one of the virulence determinants of this bacteria. T3SS1 expression is regulated by ToxR and CalR. ToxR represses the transcription of T3SS1 genes via activation of CalR, which acts as a transcriptional repressor of T3SS1 genes. However, the transcriptional regulation mechanisms have not been elucidated. As showing in the present work, ToxR binds to the promoter DNA region of *calR* to activate its transcription. CalR occupies the promoter-proximal regions of each detected target operons in T3SS1 loci to repress their transcription, and thereby inhibiting T3SS1-dependent cytotoxicity. Moreover, a feedback CalR inhibits *toxR* and its own gene in a direct manner. Collectively, this work reported an interesting gene regulatory network involving the reciprocal regulation of ToxR and CalR, and their regulation on T3SS1 genes transcription in *V. parahaemolyticus*.

## INTRODUCTION

*Vibrio parahaemolyticus,* the etiological agent of gastroenteritis, is a Gram-negative halophilic bacterium mostly found in marine and estuarine habitats. Human infections occurs through the consumption of contaminated poorly cooked or raw seafood with clinical manifestations like diarrhea, nausea, vomiting and abdominal cramping [1]. *V. parahaemolyticus* strains can also cause skin infection and septicemia if the bacterium enters into open wounds [1, 2]. *V. parahaemolyticus* has been recognized as a leading cause of seafood associated gastroenteritis [3].

The type III secretion system (T3SS), a novel needle-like bacterial protein injection machinery, is used by most Gram-negative bacteria to deliver toxic proteins called effectors into host eukaryotic cytoplasm, where they can manipulate host cell function [4]. The *V. parahaemolyticus* strain RIMD2210633 possesses two T3SS loci, named as T3SS1 (*vp1656-1702*) and T3SS2 (*vpa1321-1731*), respectively [5]. T3SS1 predominantly contributes to *V. parahaemolyticus*-induced cytotoxicity in most mammalian cell lines and lethality in the mouse infection model *in vivo* [6, 7]. T3SS1 destroy host cells through autophagy, membrane blebbing, cell-rounding and cell lysis [8, 9]. T3SS2 is related to *V. parahaemolyticus* enterotoxicity with symptoms such as diarrhea, disruption of the intestinal epithelia and inflammation [6]. In the recent years, many toxic effectors of T3SS1 and 2 that are involved in pathogenesis were identified in *V. parahaemolyticus* [7, 10–15].

ToxR is a transmembrane regulatory protein that functions in DNA binding and transcriptional regulation in pathogenic *Vibrio* species. In *V. cholerae*, disease occurs due to the role of ToxR which acts with TcpP to induce *toxT* expression [16, 17]. ToxT in turn induces expression of colonization factors and cholera toxin [17]. ToxR also can directly activate the cholera toxin genes *ctxAB* expression in the presence of bile acids, as well as some outer membrane proteins, *ompT* and *ompU* [18–20]. Kazi et al., in their recent work demonstrated that ToxR shares more than a third of its regulon with the histone-like nucleoid structuring protein H-NS, and antagonizes H-NS for control of critical colonization functions in *V. cholerae* [21]. ToxR in *V. parahaemolyticus* is well conserved with that of in *V. cholerae* [22]. Previous studies demonstrated that ToxR induces the expression of TDH (hemolytic activity), T3SS2 (enterotoxicity), and OmpU (fitness), but the molecular mechanisms have not been fully elucidated [22–24]. ToxR also represses the transcription of T3SS1 genes via activation of CalR, which acts as a repressor of T3SS1 genes [23].

*V. parahaemolyticus* CalR, a LysR-type tran-scriptional regulator, was originally described as a transcriptional repressor of swarming motility and T3SS1 expression [25]. Our recent study reported of a new physiological role for CalR as a repressor of the *tdh2* transcription, and thereby inhibiting hemolytic activity against human type O erythrocytes [26]. We also demonstrated that CalR is a positive regulator of the adhesion of *V. parahaemolyticus* to HeLa cells through direct acting on the three putative operons of T6SS2 locus, as well as a transcriptional activator of *cpsQ-mfpABC* and *mfpABC* in a direct and indirect manner, respectively [27, 28]. Expression of CalR itself is induced by cold shock, low concentration of sodium and calcium ions, and the regulatory protein ToxR [25, 29, 30].

**Figure 1:**
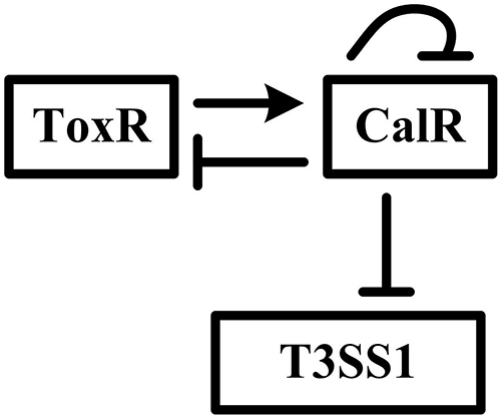
Gene regulatory circuit The details for regulatory circuit are described in the main text. The arrow line represents positive regulation. The vertical lines represents negative regulation.

In the present work, we present an interesting gene regulatory network involving the reciprocal regulation of ToxR and CalR, and negative regulation of T3SS1 and *calR* by CalR in *V. parahaemolyticus* (Figure 1). We report that ToxR has a direct and positive regulation on *calR* transcription, while CalR inhibits T3SS1 genes expression in a direct manner, and thus ToxR represses T3SS1 genes transcription via direct activation of CalR. As a feedback loop, CalR inhibits *toxR* and its own gene *calR* in a direct manner. Thus, the transcription of T3SS1 genes is tightly regulated by ToxR and CalR in *V. parahaemolyticus*.

## RESULTS

### Positive regulation of *calR* by ToxR

**Figure 2:**
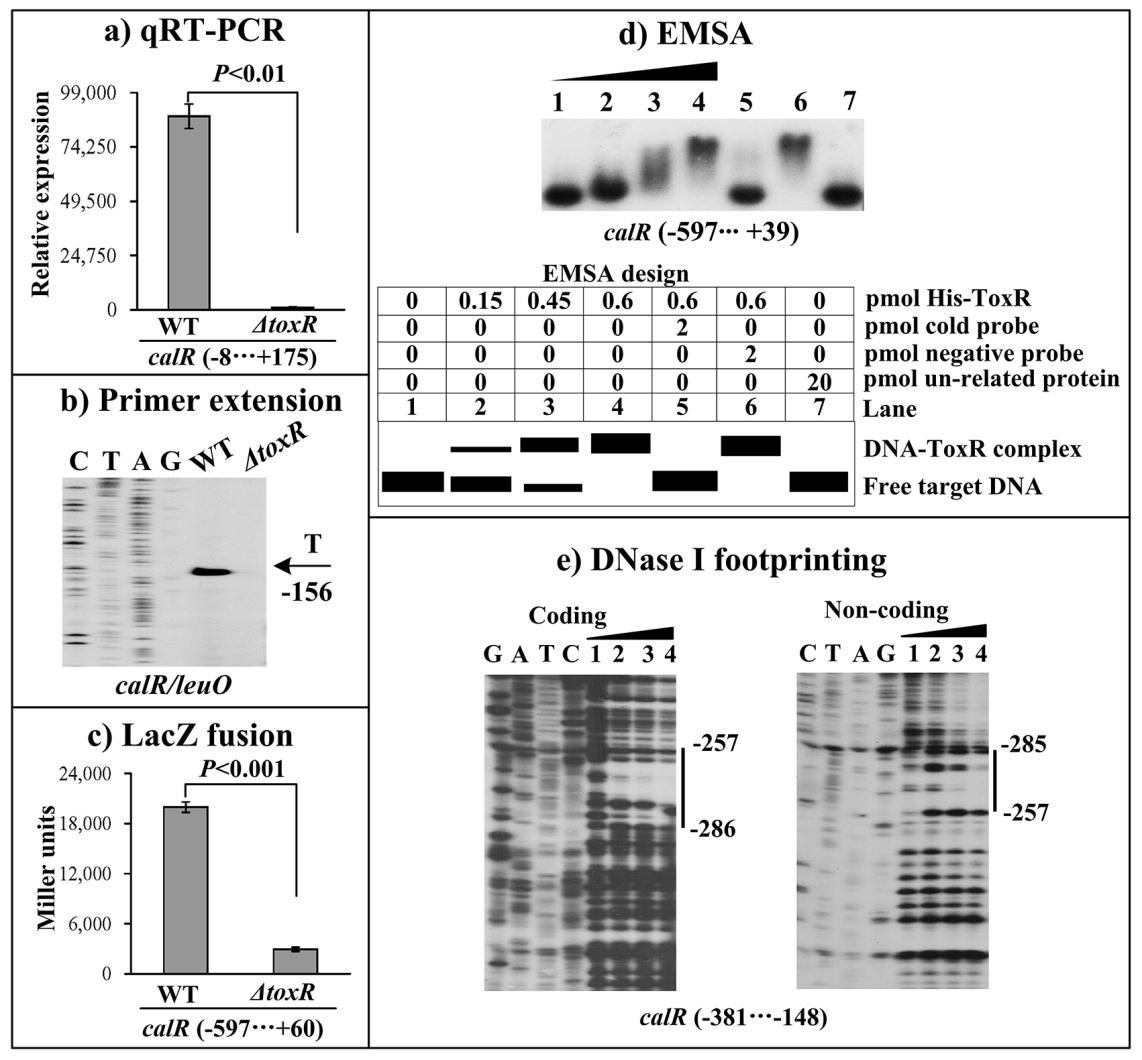
ToxR activates the transcription of *calR* **(A) qRT-PCR.** The relative mRNA level of *calR* was compared between *ΔtoxR* and WT. **(B) Primer extension.** An oligonucleotide primer was designed to be complementary to the RNA transcript of *calR*. The primer extension products were analyzed with an 8 M urea -6% acrylamide sequencing gel. The transcription start site is indicated by the arrow with nucleotide and position. **(C) LacZ fusion assay**. The entire promoter-proximal region of *toxR* was cloned into pHRP309, and then transformed into WT or *ΔtoxR* to determine the β-galactosidase activity (miller units) in cellular extracts. **(D) EMSA**. The entire promoter-proximal region of *calR* was incubated with increasing amounts of purified His-ToxR protein, and then subjected to 6% (w/v) polyacrylamide gel electrophoresis. Shown below the binding is the schematic representation of the EMSA design. **(E) DNase I footprinting.** Labeled coding or non-coding DNA probes were incubated with increasing amounts of purified His-ToxR (Lanes 1, 2, 3, and 4 containing 0, 0.2, 0.6, and 0.8 pmol, respectively), and subjected to DNase I footprinting assay. The footprint regions were indicated with vertical bars. The negative and positive numbers represent the nucleotide position upstream and downstream of *calR*, respectively. Lanes C, T, A and G represent the Sanger sequencing reactions.

The qRT-PCR assay was conducted to detect the mRNA levels of the target genes in WT and *ΔtoxR* (Figure 2A). The result indicated that the mRNA level of *calR* was greatly decreased in *ΔtoxR* relative to WT. The primer extension assay (Figure 2B) further disclosed that the mRNA level of *calR* decreased in *ΔtoxR* relative to WT. The recombinant *lacZ* fusion plasmid that contains the indicated promoter-proximal region and promoterless *lacZ* gene was transformed into WT and *ΔtoxR*, respectively, to test the action of ToxR on the promoter activity of *calR*. The results showed that the *calR* promoter activity significantly decreased in *ΔtoxR* relative to WT (Figure 2C). The entire promoter DNA regions of *calR* was amplified, purified, and subjected to EMSA with purified His-ToxR protein (Figure 2D). The result showed that His-ToxR was able to bind to the upstream DNA fragment of *calR* promoter in a dose dependent manner. As further determined by DNase I footprinting (Figure 2E), His-ToxR protected a single DNA region from 286 to 257 bp upstream of *calR* against DNase I digestion considered as the ToxR site. Taken together, ToxR activates *calR* transcription in a direct manner.

### Negative regulation of *toxR* by CalR

**Figure 3:**
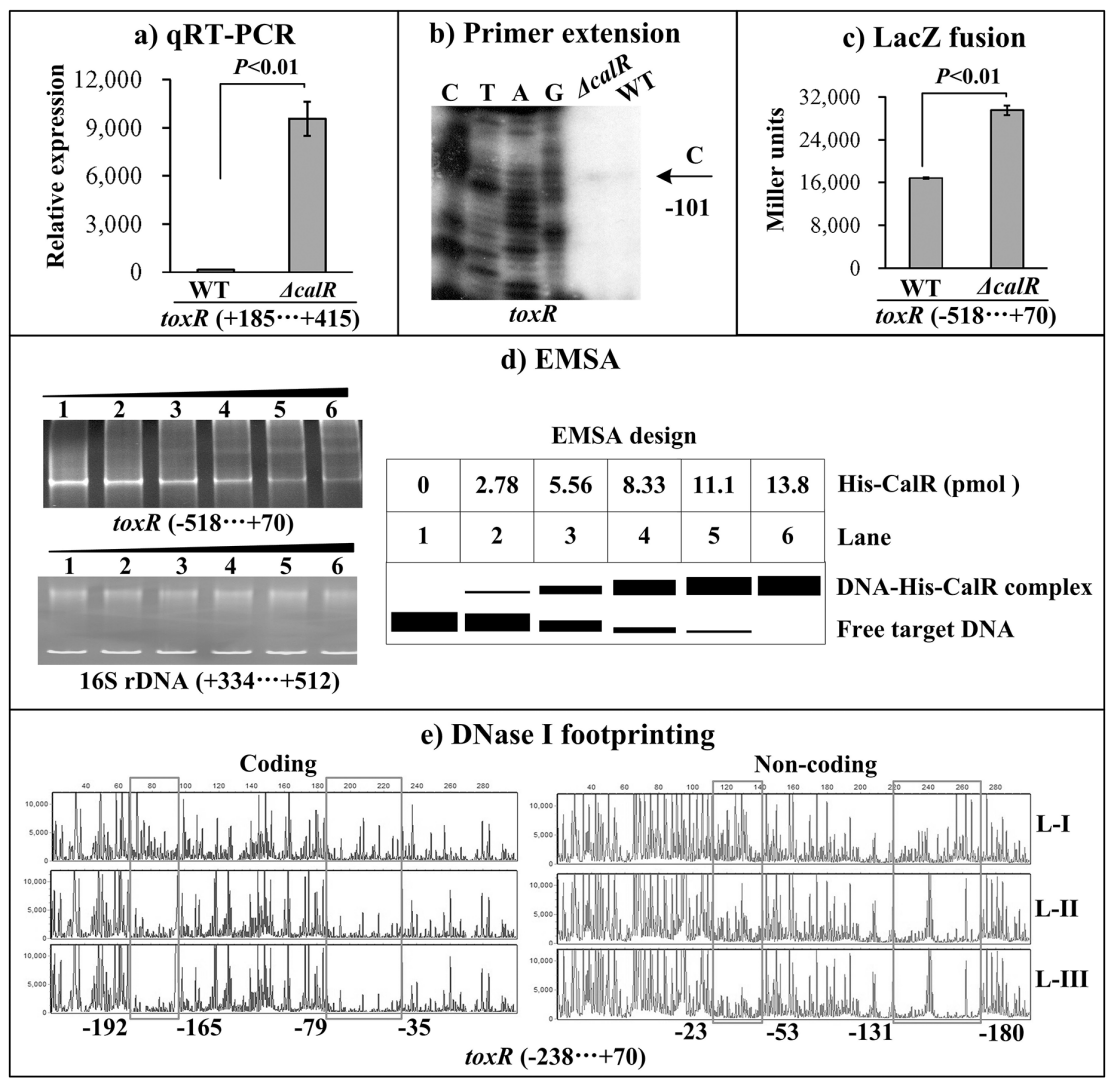
CalR represses the transcription of *toxR* The negative and positive numbers represent the nucleotide position upstream and downstream of *toxR*, respectively. **(A)** qRT-PCR, **(B)** primer extension, and **(C)** LacZ fusion were done as Figure 2. **(D)** EMSA. The promoter DNA region of *toxR* was incubated with increasing amounts of purified His-CalR, and then subjected to 6% (w/v) polyacrylamide gel electrophoresis. The DNA bands were visualized by EB staining. Shown below the EMSA results is the EMSA design. **(E)** DNase I footprinting. The promoter fragment of *toxR* was labelled with FAM or HEX, incubated with increasing amounts of purified His-CalR (Lanes-I, II, and III containing 0, 5.52, and 11.04 pmol, respectively), and then subjected to DNase I footprinting assay. The fragments length was analyzed using an ABI 3500XL DNA analyzer. The footprint regions were boxed and marked with positions.

The primer extension assay detected a single transcription start site at 101bp upstream of *toxR*, and its transcriptional activity is under the negative control of CalR (Figure 3B). The qRT-PCR and LacZ fusion assays further confirmed the negative correlation between CalR and *toxR* transcription (Figure 3A, and 3C). EMSA results showed that His-CalR was able to bind to the upstream DNA fragment of *toxR* in a dose dependent manner and the His-CalR proteins at all amounts used could not bind to the 16S rDNA fragment as the negative control (Figure 3D). As further determined by DNase I footprinting (Figure 3E), His-CalR protected two different DNA regions upstream of *toxR* against DNase I digestion that were considered as the CalR sites. Taken together, CalR represses the transcription of *toxR* in a direct manner.

### Autoregulation of CalR

**Figure 4:**
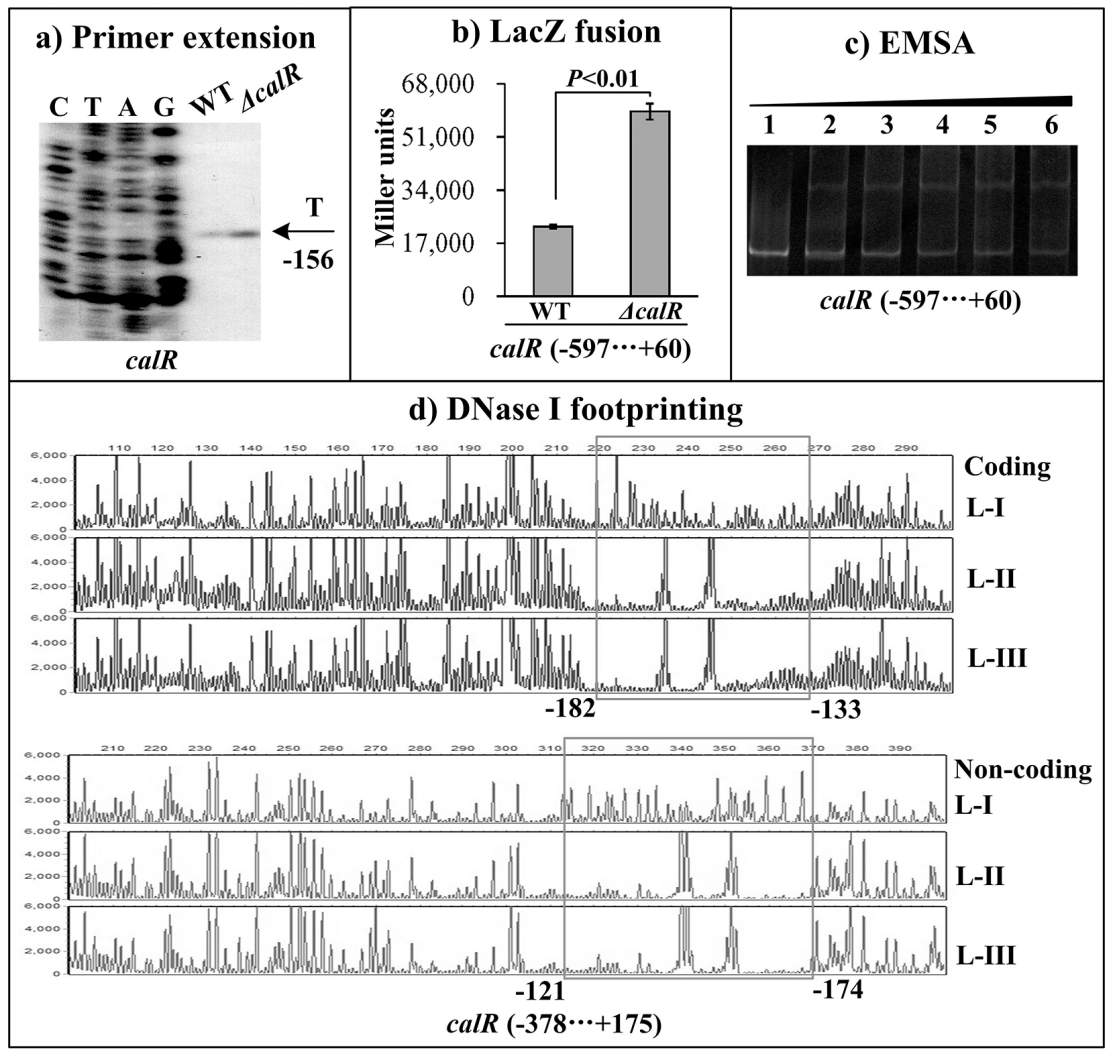
Autoregulation of *calR* The negative and positive numbers represent the nucleotide position upstream and downstream of *calR*, respectively. **(A) qRT-PCR**, **(B) primer extension**, and **(C) LacZ fusion** were done as Figure 2. **(D) EMSA** and **(E) DNase I footprinting** were done as Figure 3.

The primer extension assay detected a single transcription start site at 156 bp upstream of *calR* which was under the negative control of CalR (Figure 4A). The *lacZ* fusion result showed that the promoter activity of *calR* is significantly enhanced in *ΔcalR* than that in WT (Figure 4B), suggesting a negative correlation between CalR and its own gene transcription. The EMSA and DNase I footprinting assays were conducted to investigate the binding activity of CalR to its own promoter DNA. The results showed that His-CalR protects a single DNA region from 121 bp to 182 bp upstream of *calR* against DNase I digestion in a dose dependent manner (Figure 4C, and 4D). Thus, these results indicated the direct binding activity and the negative autoregulation of CalR.

### CalR represses the cytotoxic activity against HeLa cells

**Figure 5:**
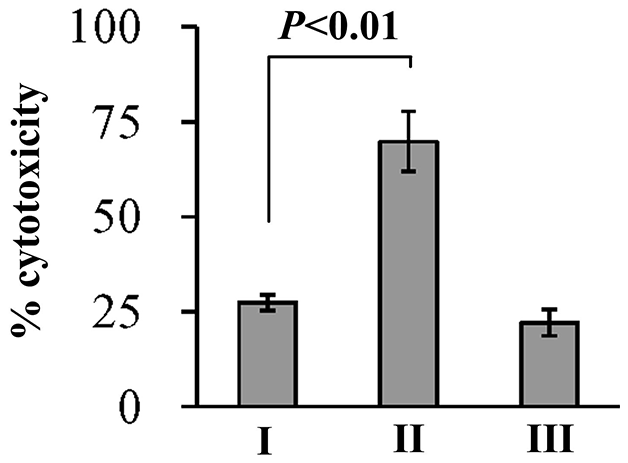
Cytotoxicity against HeLa cells of *V. parahaemolyticus* strains HeLa cells were infected with *V. parahaemolyticus* strains at [MOI] =1:2.5. The percentage cytotoxicity was calculated as HeLa cells killed/input bacterial cells. I, II, and III represent WT/pBDA33, *ΔcalR*/pBDA33 and *ΔcalR*/pBDA33-*calR*, respectively.

*V. parahaemolyticus* cytotoxic activity against the HeLa cells was evaluated in terms of the release of LDH from cultured cells (Figure 5). The cytotoxicity against the HeLa cells infected with *ΔcalR*/pBDA33 was significantly increased than that of WT/pBDA33 or c-*ΔcalR*, while there was no significant change between *ΔcalR/*pBAD33-*calR* and WT */*pBAD33. These results confirmed that *V. parahaemolyticus* CalR acts as a repressor of the cytotoxic activity against HeLa cells.

### Negative regulation of T3SS1 genes by CalR

**Figure 6:**
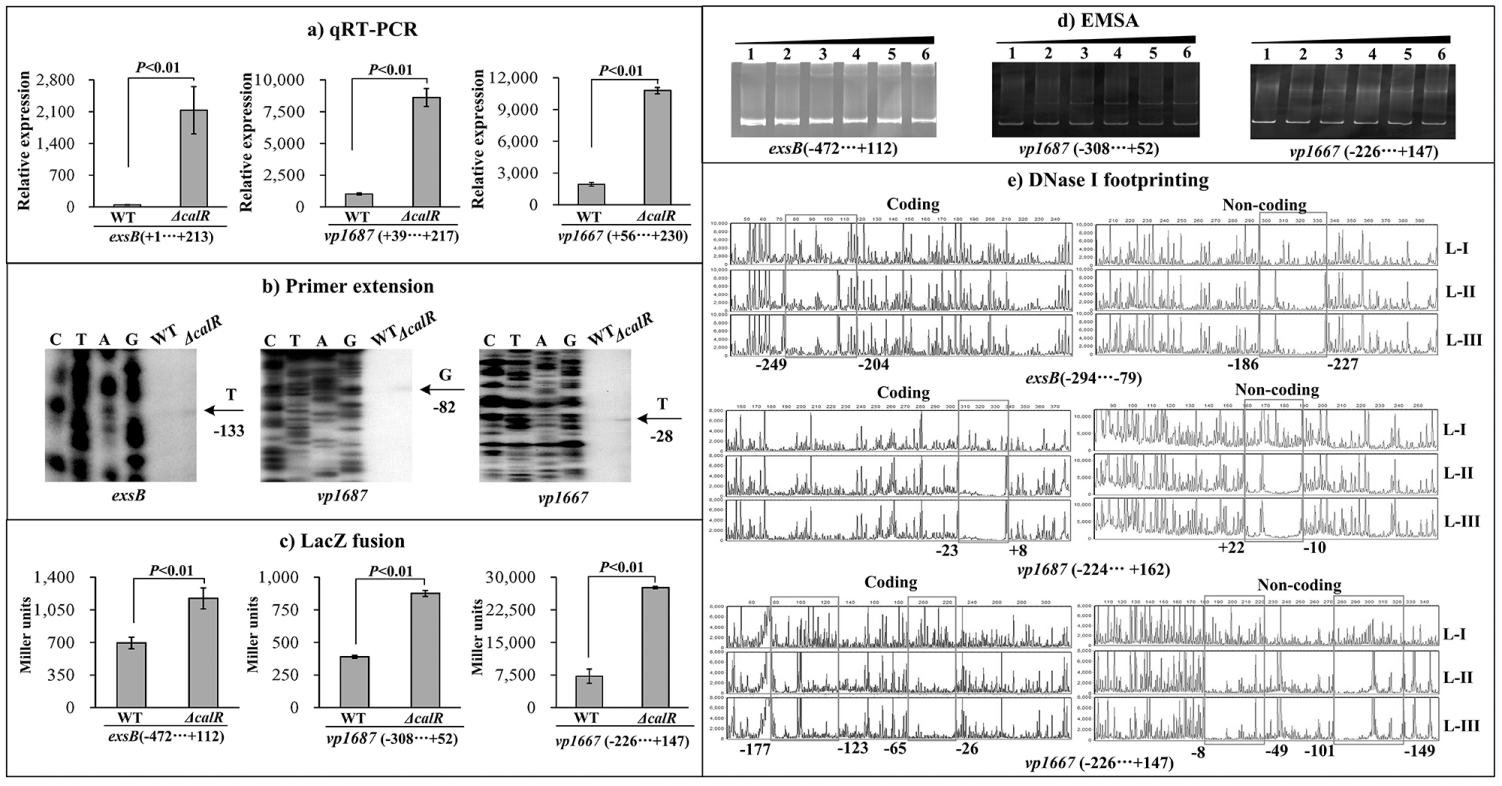
CalR represses the transcription of T3SS1 genes **(A)** qRT-PCR, **(B)** primer extension, and **(C)** LacZ fusion were done as Figure 2. **(D)** EMSA and **(E) DNase** I footprinting were done as Figure 3.

T3SS1 locus is composed of at least ten putative operons. The three operons *vp1700-1688* (*exsBAD-vscBCD*), *vp1667-1655* and *vp1687-1686* were arbitrarily selected, and the first genes of each of the operons were subjected to the following investigations: qRT-PCR, primer extension, LacZ fusion, EMSA and DNase I footprinting. The qRT-PCR results indicated that the mRNA level of each target gene was greatly increased in *ΔcalR* relative to WT (Figure 6A). The primer extension assay disclosed a single transcription start site for each of the three operons, and their transcription activities were under the negative control of CalR (Figure 6B). The *lacZ* fusion results showed that the promoter activity of each of the three operons in *ΔcalR* was much higher relative to that in WT (Figure 6C). EMSA results showed that His-CalR was able to bind to each of the target DNA fragment in a dose-dependent manner (Figure 6D). As further determined by DNase I footprinting (Figure 6E), His-CalR protected two different DNA regions upstream of *vp1667* against DNase I digestion that were considered as the CalR sites; however, a single DNA region upstream was protected against DNase digestion in both *exsB* and *vp1687*. The above results revealed the negative regulation of each target gene by CalR, respectively.

### Promoter structure of target genes

**Figure 7:**
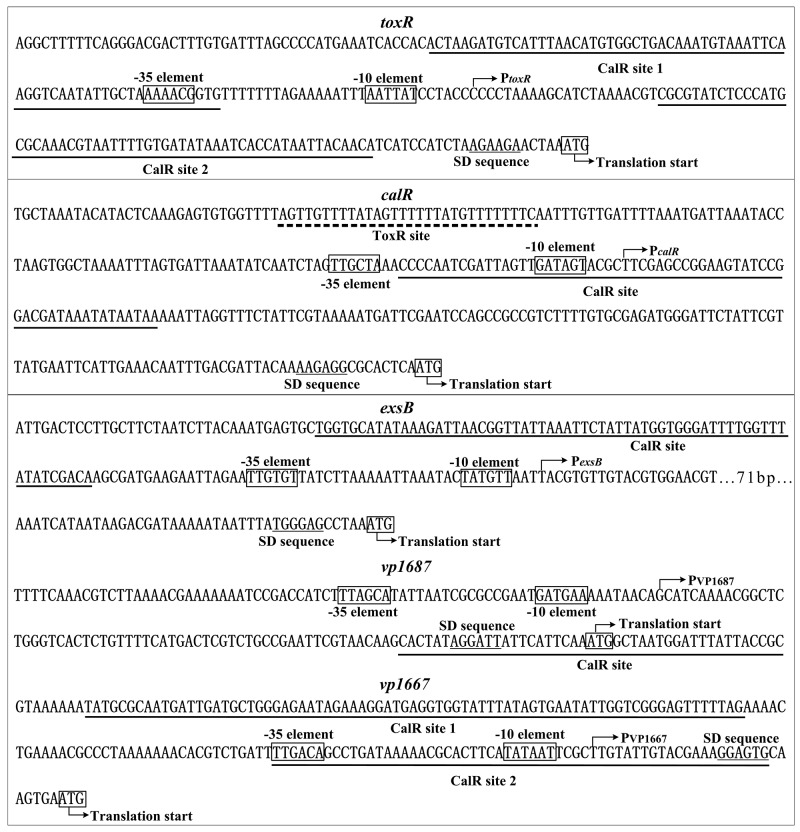
Structural organization of target promote rs The DNA sequence was derived from RIMD 221063. The transcription start site is indicated by bent arrows. The SD box and -10/-35 elements are enclosed in boxes. The CalR sites are underlined with solid lines, while the ToxR site is underlined with a broken line.

The primer extension assays detected a single transcriptional start site for each target gene, through which we determined the corresponding -10 and -35 elements. The DNase I footprinting assays identified one or more footprint sites for each target promoter-proximal region, which is considered as the ToxR or CalR binding sites. Thus, we depicted the structural organization of the promoter of *toxR, calR*, *exsB, vp1687* and *vp1667* by collecting the data of the translation/transcription start sites, promoter -10 and -35 elements, CalR sites, and Shine-Dalgarno (SD) sequences (ribosomal binding sites) (Figure 7).

## DISCUSSION

T3SSs are tightly regulated protein secretion systems that are used by most Gram-negative bacteria to deliver toxic effectors into host eukaryotic cytoplasm [4]. T3SS gene expression is sophisticatedly regulated by the transcriptional regulatory system ExsACDE. In *Pseudomonas aeruginosa*, ExsA is a positive regulator of T3SS expression [31]. ExsD acts as an anti-activator by complexing with ExsA, which prevents ExsA fuction [31]. ExsC is an anti-anti-activator that binds ExsD to prevent the ExsD-ExsA complex formation, thereby allowing ExsA to activate T3SS gene expression [31]. ExsE is a secreted substrate of the T3SS that binds ExsC and antagonizes the regulatory activity of ExsC [31]. The expression of T3SS1 genes in *V. parahaemolyticus* is similar to the ExsACDE regulatory cascade in *P. aeruginosa* [32, 33]. Sun and his colleagues previously reported that H-NS binds to the promoter regions of *exsA*, *exsC*, and *exsD* to repress their transcription, and thereby inhibiting T3SS1 expression and *V. parahaemolyticus*-induced cytotoxicity [34, 35]. High level of T3SS1 expression occurs by growing bacteria in high-calcium or low-iron growth conditions [25]. LafK, a key σ^54^-dependent regulator, appears to be required to mediate the major portion of the T3SS1 response to high-calcium [25]. By contrast, CalR, which negatively regulates T3SS1 transcription, is required for the low calcium-mediated decrease of T3SS1 gene expression [25]. Although the regulatory mechanisms were not clarified, Whitaker et al., demonstrated that ToxR inhibits the expression of T3SS1 genes via positive regulation of *calR* [23]. In addition, the quorum sensing system in *V. parahaemolyticus* appears to activate and repress T3SS1 regulons at low-cell density and high-cell density, respectively [36, 37]. Moreover, the small RNA Spot 42 is involved in regulating the expression of T3SS1 gene *vp1682* at post-transcriptional level in *V. parahaemolyticus*, which contributes to cytotoxicity in *vivo* [38].

The present work demonstrates that *V. parahaemolyticus* ToxR binds to the promoter DNA region from 286 to 257 bp upstream of *calR* to activate its transcription. CalR occupies the promoter-proximal regions of the three operons (*exsBAD-vscBCD*, *vp1687-1686*, and *vp1667-1655*) in T3SS1 locus to repress their transcription, thereby inhibiting T3SS1-dependent cytotoxicity phenotype. Thus, ToxR inhibits T3SS1 expression via direct activation of *calR*. As a feedback loop, CalR binds to the promoter-proximal regions of *toxR* and its own gene to inhibit their transcription. Thus, we report an interesting gene regulatory network involving the reciprocal negative regulation of ToxR and CalR, and negative regulation of T3SS1 and *calR* by CalR in *V. parahaemolyticus*.

The structural organization of each target promoter was reconstructed based on the collection of translation/transcription start sites, -10 and -35 elements, SD sequences, and ToxR or/and CalR binding sites (Figure 7). The ToxR site for *calR* is far upstream of the -35 element, and thus the ToxR-dependent *calR* promoter may belong to class I transcriptional stimulation [39]. The CalR sites for *calR* and *vp1687* overlaps or downstream of the -10 element, the binding of CalR to the *calR* or *vp1687* promoter region would block the entry or elongation of RNA polymerase (RNAP), and a similar mechanism has been observed for *Yersinia pestis* PhoP [40]. CalR bound to two sites within the upstream region of the *toxR* and *vp1667*, and one of the two sites for each gene overlaps or downstream of the transcriptional start site, these two binding sites would form a hairpin in the promoter and thus block the entry or elongation of RNAP. The CalR site for *exsB* is located upstream of the -35 element, which is highly unusual for transcriptional repression. However, a similar mechanism has been observed for CalR inhibition of *tdh2* in *V. parahaemolyticus* [26], and there would be a potential transcriptional activator for *exsB* transcription. Overall, *V. parahaemolyticus* CalR uses a variety of mechanisms to regulate the transcription of its target genes. Nevertheless, whether ToxR have direct regulation on its own gene and T3SS1 genes need to be further characterized.

## MATERIALS AND METHODS

### Bacterial strains and plasmids

The *V. parahaemolyticus* strain RIMD2210633 was used as the wild type (WT) in this study [5]. The *calR* deletion mutant (*ΔcalR*) was generated as previously described [41, 42]. Briefly, the 405 and 426 bp DNA regions upstream and downstream of *calR* were amplified by PCR using primers *calR*-A and -B, and *calR*-C and -D, respectively. After being purified, the amplicons were used as the templates to create an 801 bp deletion construct using primers *calR*-A and -D, which was subsequently inserted between the *Pst*I and *Sph*I sites of the pDS132 containing the *sacB* gene conferring sensitivity to sucrose, and a chloramphenicol resistance gene. After being verified by DNA sequencing, the recombinant vector was transformed into *Escherichia coli* S17-1(λpir), and then transferred into WT by conjugation. The *ΔcalR* was selected using resistance to 10% sucrose and sensitivity to 5 μg/ml chloramphenicol, and further verified by PCR. Using the same method, the entire coding region of *toxR* was deleted from WT genome to create *toxR* deletion mutant (*ΔtoxR*). All the primers used in the present work were listed in Table 1.

**Table 1:** Oligonucleotide primers used in this study

Type of analysis and primer	Sequences (5′-3′)
**Construction of mutants**	
*calR*-A	GTAGCTGCAGGCAGATTATTTGACTGATACGC
*calR*-B	GTTCGCAAATGGGAAGTCTCTCATCGCATCTTTCTTCTC
*calR*-C	GAGAAGAAAGATGCGATGAGAGACTTCCCATTTGCGAAC
*calR*-D	GTGAGCATGCTACTTACCTTTTGGCTTACAG
*toxR-*A	GTGACTGCAGAAACGCAATTTGTCTGATG
*toxR-*B	ATCTTCATGCTGGCCTCCTTTAGTTCTTCTTAGATGGATGATG
*toxR-*C	CATCATCCATCTAAGAAGAACTAAAGGAGGCCAGCATGAAGAT
*toxR-*D	GTGAGCATGCAATTCGGCGGCTTTGTTC
**Construction of complementary strain**	
*calR*-HP-F	GCGGTCGACAGGAGGAATTCACCATGTTAGAGAAGAAAGATG
*calR*-HP-R	GCGAAGCTTTTATTTTGATGCGACCAC
*toxR-*HP-F	GATTCTAGAAGGAGGAATTCACCATGACTAACATCGGCACCAA
*toxR-*HP-R	GACAAGCTTTTATTTGCAGATGTCTGTTGG
**Protein expression**	
*calR*-P-F	GCGGGATCCATGTTAGAGAAGAAAGATG
*calR*-P-R	GCGAAGCTTTTATTTTGATGCGACCAC
*toxR-*P-F	AGCGGGATCCATGACTAACATCGGCACCAA
*toxR-*P-R	AGCGAAGCTTTTAAGGATTCACAGCAGAAG
**qRT-PCR**	
*calR*-RT-F	ATGTAAAAAGAAAACCGTACA
*calR*-RT-R	AACACAGCAGAATGACCGTG
*toxR-*RT-F	TTGTTTGGCGTGAGCAAGG
*toxR-*RT-R	TAGCAGAGGCGTCATTGTTATC
*exsB-*RT-F	ATGAAAAGCAGTAAGTGGGC
*exsB-*RT-R	CTGAGAAGCAACAGTAAGAC
*vp1687-*RT-F	TGCTCACCGTTGCCAAATAG
*vp1687-*RT-R	GCGACGCTTTCATGTATTGC
*vp1667-*RT-R	GGAATGGATTGGAATCGTC
*vp1667-*RT-R	CCACCGTCTTTTATTTTGC
16S rDNA-RT-F	GACACGGTCCAGACTCCTAC
16S rDNA-RT-R	GGTGCTTCTTCTGTCGCTAAC
**Primer extension**	
*calR*-PE-R	GCAAAATATCGGTACTTCA
*toxR-*PE-R	TTAGTTCTTCTTAGATGGATGATG
*exsB-*PE-R	GTCTTATTATGATTTATTTTTACAC
*vp1687-*PE-R	GGCAACGGTGAGCAAAATC
*vp1667-*PE-R	GACGATTCCAATCCATTCCG
**LacZ fusion**	
*calR-lacZ*-F	GCGGTCGACGTTTGTTTGCTCGGATTGTTTG
*calR-lacZ*-R	GCGTCTAGACAAAGTGCTTTCCATACGGTAG
*toxR-lacZ*-F	GCGCGTCGACATCGTTAAGGTATTTGCA
*toxR-lacZ*-R	GCGCGAATTCCGAGCGAATTACTATTTGG
*exsB-lacZ*-F	ATATGTCGACATTGTCCGTCAAATGCAGTTC
*exsB-lacZ*-R	TTTTGAATTC CATATACATTCGCTTGGCTCTG
*vp1687-lacZ*-F	GCGCGTCGACGCATTATTGACGCCAGTATCG
*vp1687-lacZ*-R	GCGCTCTAGAGGCAACGGTGAGCAAAATC
*vp1667-lacZ*-F	GCGGTCGACCAGATTGCTGAATATCGGTG
*vp1667-lacZ*-R	GCGTCTAGA AAGCGATTGAGTGGCGTTG
**EMSA**	
*calR-*EMSA-F1	GTTTGTTTGCTCGGATTGTTTG
*calR-*EMSA-R	CAAAGTGCTTTCCATACGGTAG
*toxR-*EMSA-F	ATCGTTAAGGTATTTGCA
*toxR-*EMSA-R	CGAGCGAATTACTATTTGG
*exsB-*EMSA-F	ATTGTCCGTCAAATGCAGTTC
*exsB-*EMSA-R	CATATACATTCGCTTGGCTCTG
*vp1687-*EMSA-F	GCATTATTGACGCCAGTATCG
*vp1687-*EMSA-R	GGCAACGGTGAGCAAAATC
*vp1667-*EMSA-F	CAGATTGCTGAATATCGGTG
*vp1667-*EMSA-R	AAGCGATTGAGTGGCGTTG
**DNase I footprinting**	
*calR*-FP-F	CAGATTGCTGAATATCGGTG
*calR*-FP-R	ATTGATAATACTCATTCACTTGC
*calR*-FP-F (M13F)	GTAAAACGACGGCCAGTCCGTTGGTTATTGATAG
*calR*-FP-R (M13R)	CAGGAAACAGCTATGACCCACGGCATTACTTACTG
*toxR*-FP-F (M13F)	GTAAAACGACGGCCAGTTTTCAGGGACGACTTTGTG
*toxR*-FP-R (M13R)	CAGGAAACAGCTATGACCGAGCGAATTACTATTTGG
*exsB*-FP-F (M13F)	GTAAAACGACGGCCAGTGTTTATCAATTTTGGTTGTTAG
*exsB*-FP-R (M13R)	CAGGAAACAGCTATGACCGGCTTATATTTATTCTAC
*vp1687*-FP-F (M13F)	GTAAAACGACGGCCAGTCACCAGAGTAGGGCATCAC
*vp1687*-FP-R (M13R)	CAGGAAACAGCTATGACCAAGCCAATGAGCGTCAG
*vp1667*-FP-F (M13F)	GTAAAACGACGGCCAGTCAGATTGCTGAATATCGGTG
*vp1667*-FP-R (M13R)	CAGGAAACAGCTATGACAAGCGATTGAGTGGCGTTG
M13F-FAM	GTAAAACGACGGCCAGT
M13R-HEX	CAGGAAACAGCTATGAC

To complement the *ΔcalR* or *ΔtoxR* [35], a PCR-generated DNA fragment containing the *calR* or *toxR* coding region together with an upstream synthetic SD sequence (AGGAGG) was inserted between the *Sal* I and *Hind* III sites of the vector pBAD33 harboring an arabinose P_BAD_ promoter and a chloramphenicol resistance gene.After beingverified by DNA sequencing, the recombinant pBAD33-*calR or* pBAD33*-toxR* plasmid was transformed into *ΔcalR or ΔtoxR*, yielding the complemented mutant strain *ΔcalR/*pBAD33-*calR or ΔtoxR/*pBAD33-*toxR*. For controls, the empty vector pBAD33 was also transformed into WT and *ΔcalR* or *ΔtoxR* to generate WT/pBAD33 and *ΔcalR/*pBAD33 or *ΔtoxR/*pBAD33.

### Bacterial growth conditions

*V. parahaemolyticus* was cultured in complete HI broth containing 2.5% Bacto heart infusion (BD Bioscience) at 37 °C with shaking at 250 rpm. The glyceric stock of bacterial cells were inoculated into 5 ml of HI broth and incubated overnight for at least over 14h. The overnight cell cultures were diluted 1: 50 into 15 ml of fresh HI broth, and grown to reach at OD_600_ ≈ 1.0 to 1.2, and then diluted 1:1000 into 15 ml of HI broth for the third-round growth, and were harvested at an OD_600_ value of about 1.0 to 1.2. When required, the culture medium was supplemented with 50 μg/ml gentamicin, 5 μg/ml chloramphenicol, or 0.1% arabinose.

### RNA isolation and quantitative real-time PCR (qRT-PCR)

Total RNAs were extracted using the TRIzol reagent (Invitrogen). RNA quality and quantity were monitored by agarose gel electrophoresis and spectrophotometry, respectively [41, 42]. The contaminated genome DNA in the total RNAs was removed by using the Ambion’s DNA-free^TM^ Kit. cDNAs were generated by using 3-8μg of RNA and 3μg of random hexamer primers. The SYBR Green qRT-PCR assay was performed and analyzed as previously described [43]. The experiment was performed with at least three independent cultures and RNA preparations.

### Primer extension assay

For the primer extension assay [41, 42], 3-10 μg of total RNAs was annealed with 1 pmol of 5’- ^32^P-labeled reverse oligonucleotide primer to generate cDNAs using a Primer Extension System (Promega) according to the manufacturer’s instructions. The same labeled primer was used for sequencing with the AccuPower & Top DNA Sequencing Kit (Bioneer). The primer extension products and sequencing materials were concentrated and analyzed in an 8M urea-6% polyacrylamide gel electrophoresis, and the results were detected by autoradiography with the Fuji Medical X-ray film.

### LacZ fusion and β-galactosidase assay

The promoter DNA region of each indicated gene was amplified and cloned into the corresponding restriction endonuclease sites of low-copy-number plasmid pHRP309 that harbors a gentamicin resistance gene and a promoterless *lacZ* reporter gene [44]. After being verified by DNA sequencing, the recombinant pHRP309 plasmid was transferred into *V. parahaemolyticus* strains. An empty pHRP309 plasmid was also introduced into each strain tested as the negative control. The *V. parahaemolyticus* strains transformed with recombinant or empty pHRP309 plasmids were grown as above to measure the β-galactosidase activity in cellular extracts using the β-Galactosidase Enzyme Assay System (Promega) according to the manufacturer’s instructions.

### Preparation of 6× His-tagged CalR (His-CalR) and (His-ToxR) proteins

The entire coding region of *calR* or the truncated *toxR* (1-528 bp, a.a.1-176) was amplified, purified, and cloned into pET28a (Novagen). The recombinant plasmid encoding His-CalR or His-ToxR was then transformed into *E. coli* BL21λDE3 cells. Expression and purification of His-CalR were similar to that of His-OpaR[41], while His-ToxR was the same as that of His-AphA [42]. The purified proteins were concentrated with nickel loaded HiTrap Chelating Sepharose columns (Amersham) and concentrated to a final concentration of about 0.3-0.6 mg/ml. The purified proteins were stored at -80°C, and the protein purity was confirmed by SDS-PAGE.

### Electrophoretic mobility shift assay (EMSA)

EMSA was performed with two methods according to the difference in raw material: the radioactive labeled probe and ethidium bromide (EB) stain method [26, 41]. For the EB stain method [26], DNA binding was performed in a 10 μl reaction volume containing binding buffer [1 mM MgCl_2_, 0.5 mM EDTA, 0.5 mM DTT, 50 mM NaCl, 10 mM Tris-HCl (pH 7.5) and 10 mg/ml salmon sperm DNA], 100-200 ng target promoter DNA, and increasing amounts of His-CalR. After incubation at room temperature for 20 min, the products were loaded onto a native 6 % (w/v) polyacrylamide gel, and electrophoresed in 0.5× TBE buffer for about 90 min at 200 V. The gel was examined with a UV transilluminator after staining with the EB dye. For the radioactive labeled probe method [41], the 5’ ends of target DNA fragments were labeled using [γ-^32^P] ATP and T4 polynucleotide kinase. DNA binding was also performed in a 10 μl reaction volume containing binding buffer, radio-labeled DNA, and increasing amounts of His-ToxR. Three controls were included in each EMSA experiment: 1) cold probe as specific DNA competitor (the same promoter-proximal DNA region unlabeled), 2) negative probe as non-specific DNA competitor (the unlabeled coding region of the 16S rRNA gene), and 3) a non-specific protein competitor (rabbit anti-F1-protein polyclonal antibodies). Radioactive species were detected by autoradiography after exposure to Fuji Medical X-ray film.

### DNase I footprinting assay

DNase I footprinting assays were performed with two different methods: the radioactive labeled probe and DNA sequencing methods. For the radioactive labeled probe method [41, 42], the promoter-proximal DNA region with a single 32P-labeled end was PCR amplified with either sense or antisense end-labeled primers. The PCR products were purified using QiaQuick columns (Qiagen). Increasing amounts of His-ToxR were incubated with the purified and labeled DNA fragments (2–5 pmol) for 30 min at room temperature. The final reaction volume was 10 μl and contained binding buffer used in EMSA. Before DNA digestion, 10 μl of Ca2+/Mg2+ solution (5 mM CaCl2 and 10 mM MgCl2) was added, followed by incubation for 1 min at room temperature. The optimized RQ1 RNase-Free DNase I (Promega) was then added to the reaction mixture, and the mixture was incubated at room temperature for 40–90 s. The reaction was quenched by adding 9 μl of stop solution (200 mM NaCl, 30 mM EDTA, and 1% SDS), followed by incubation for 1 min at room temperature. The partially digested DNA samples were then extracted with phenol/chloroform, precipitated with ethanol, and analyzed in 6% polyacrylamide/8 M urea gels. Protected regions were identified by comparison with sequencing ladders. The templates for these sequencing ladders were the same as the DNA fragments for DNase I footprinting. Radioactive species were detected by autoradiography.

For the DNA sequencing method [26, 28], a DNA fragment of promoter DNA region of each indicated genes was PCR amplified using the primers target-FP-F (M13F) and target-FP-R (M13R) with *ExTaq* DNA ploymerase. After being purified, the amplicon was used as the template for labeling the probes with different primer pairs: M13F-FAM and target gene-FP-R (M13R) for preparation of 6-carboxyfluorescein (FAM)-labeled coding strand, and target gene-FP-F (M13F) and M13R-HEX for preparation of 5’-Hexachlorofluorescein phosphoramidite (HEX)-labeled noncoding strand. The PCR products were purified by using the Qiaquick columns (Qiagen) and quantified with a NanoDrop 2000 (Thermo). Approximately 350 ng of the purified, FAM/HEX-labeled DNA fragments were incubated with increasing amounts of His-CalR in a final 10 μl reaction volume containing the binding buffer used in EMSA. The DNA digestion procedure was the same as the radioactive labeled probe method. The digested DNA samples were extracted with a Beaver Beads ™PCR Purification Kit (Beaver) according to the manufacturer’s instructions, and the sample pellets were dissolved in 15μl modified water (HiDi: water: 600 LIZ=90: 60: 1). For sequencing, the BigDye® Terminator v3.1 Cycle Sequencing Kits (ABI) was used. The volume of each sequencing reaction was increased to 20 μl that contains 10 ng of target promoter region as template, 3.2 pmol of sense or antisense primer as the sequencing primer, and 8 μl of BigDye reaction mix (BigDye: 5× buffer = 1: 3). After pre-denaturation at 96°C for 1 min, PCR amplification was conducted at 25 cycles of denaturation at 96°C for 10 s, annealing at 50°C for 5 s and extension at 60°C for 4 min. The sequencing samples were precipitated with the same method as above, and then dissolved in 10 μl HiDi and 1μl 600 LIZ. The digested DNA fragments were analyzed by using ABI 3500XL DNA Genetic analyzer with GeneMarker software 2.2. The sequencing products were examined with Sequence Scanner software v1.0.

### Cell culture and cytotoxicity assay

HeLa cell was maintained in DMEM (Invitrogen) containing 10% fetal bovine serum (Invitrogen) at 37°C in 5% CO_2_. The cytotoxic assays were performed as described previously [6, 35]. The precultivated bacterial cells were washed and serially ten-fold diluted with the pre-warmed Dulbecco’s modified Eagle’s medium (DMEM) lacking phenol red for CFU measurement and infection. HeLa cells were infected with 10^6^ CFU of bacteria for 3h at a multiplicity of infection (MOI) of 2.5. After infection, the release of lactate dehydrogenase (LDH) into the medium was quantified with a CytoTox96 kit (Promega) according to the manufacturer’s instructions.

### Experimental replicates and statistical methods

The LacZ fusion, qRT-PCR and cytotoxicity assays were performed with at least three independent bacterial cultures and the values were expressed as mean ± standard deviation. Paired Student’s *t-*test was used to calculate statistically significant differences, *p* <0.01 was considered to indicate statistical significance. The presented data of primer extension, DNase I footprinting and EMSA assays were done with at least two independent biological replicates.
